# Special Issue “Role of Exercises in Musculoskeletal Disorders—5th Edition”

**DOI:** 10.3390/jfmk10010043

**Published:** 2025-01-22

**Authors:** Giuseppe Musumeci

**Affiliations:** 1 Department of Biomedical and Biotechnological Sciences, Section of Anatomy, Histology and Movement Science, School of Medicine, University of Catania, 95123 Catania, Italy; giuseppe.musumeci@unict.it; 2 Research Center on Motor Activities (CRAM), University of Catania, 95123 Catania, Italy

## 1. Introduction

The fifth edition of the Special Issue entitled “Role of Exercises in Musculoskeletal Disorders” has been concluded and has found considerable success with fourteen papers published. This Special Issue makes an important contribution to the discussion of implementing exercise into treatment plans by presenting innovative research and useful techniques meant to improve the physical and mental health of those who suffer from these conditions. Musculoskeletal disorders, ranging from arthritis to back pain and resulting from many other conditions such as multiple sclerosis [1] and osteoporosis, often lead to chronic musculoskeletal pain, reduced mobility, and decreased quality of life [2]. Today, regular exercise is considered essential to manage and minimize these symptoms [3,4]. Structured exercise programs help preserve joint function, enhance muscular strength, improve balance, reduce fatigue, and promote mental wellbeing [5]. However, bridging the gap between clinical evidence and practice requires further study. A morphological study of Italian students found that more than half had musculoskeletal problems due to incorrect posture and inactivity, highlighting the need for early intervention and tailored exercise programs [6]. In this context, the role of exercise extends beyond its physical benefits. It serves as a tool for enhancing social interaction [7], mitigating stress, and fostering overall life satisfaction. This evidence highlights the need for a comprehensive approach to developing tailored exercise programs that address the specific needs of patients with musculoskeletal disorders. In this way, practitioners can reduce the risks associated with sedentary lifestyles and help patients regain control over their health and wellbeing. By examining various viewpoints on the function of exercise in musculoskeletal illnesses, this Special Issue aims to address these significant topics. It explores how exercise promotes musculoskeletal health, assesses short- and long-term results, and highlights creative methods for improving patient treatment through original research and review papers. The papers in this Special Issue aim to offer useful information to patients, researchers, and healthcare providers, opening the door to more durable and effective exercise-assisted therapies.

## 2. Overview of the Published Articles

This collection of studies explores musculoskeletal disorders according to age, gender, and the sport played. It also examines the role of exercise in managing these disorders, highlighting its contributions to improving both physical and mental health. The following are the main insights from this edition of “Role of Exercises in Musculoskeletal Disorders”. The first paper published in this Special Issue is an analysis of 309 patients [8] that revealed age- and gender-related differences in the morphology of cuff tear arthropathy (CTA). Women had higher levels of fat infiltration, while older patients showed greater muscle retraction and reduced acromiohumeral distance. These morphological differences may influence clinical decisions and therapeutic approaches for managing CTA. One study [9] examined the relationship between abnormal gait patterns and physical activity levels in patients with knee osteoarthritis. Using a scoring system based on seven criteria, researchers found that an abnormal gait is a strong predictor of low physical activity levels after one year. Higher scores on the gait analysis correlated with fewer daily steps, suggesting that gait analysis could help identify patients at risk of prolonged physical inactivity. Another article [10] investigated muscle activation during bilateral biceps curls performed with a straight bar versus an EZ bar and with or without arm flexion. The results showed that the straight bar slightly increased biceps activation compared to the EZ bar, and arm flexion influenced muscle activation differently. These findings suggest that varying curl techniques can optimize neural and mechanical stimulation during training. The COVID-19 pandemic has had a major impact on the psychological wellbeing of athletes and perceived fatigue. A study of 204 Italian athletes [11] found that women and endurance athletes reported higher levels of fatigue and post-COVID symptoms than men and anaerobic athletes. These findings emphasize the importance of personalized medical monitoring and training programs for athletes returning to activity after infection. It has also been seen that the site of spinal cord injury affects cardiac autonomic regulation [12]. Patients with cervical lesions showed greater sympathovagal dysfunction than those with thoracic lesions, although the baroreceptor reflexes remained intact. Analysis of cardiovascular variability proved to be a valuable tool for assessing autonomic alterations, underscoring the need for further studies to refine therapeutic strategies. In the context of metabolic health, a study on female university students [13] showed that normal-weight obesity is associated with reduced skeletal muscle mass and low physical activity levels, with significant risks for sarcopenia and metabolic diseases. Promoting physical activity among young people is crucial for mitigating these long-term risks. Electrically induced low-force exercise (LFE) has shown promise in managing postprandial glucose and insulin markers in individuals with spinal cord injuries [14]. LFE significantly reduced glucose and insulin levels while increasing lactate, highlighting its potential as a metabolic management strategy for those with limited exercise options. Another study [15] examined the effects of artificially induced breast volume on neck and trunk muscle activity. Excessively large breasts significantly altered the muscle activity and could contribute to biomechanical dysfunctions, emphasizing the importance of individualized assessments in clinical care. Sedentary behavior, a growing concern in modern society, has been identified as a major barrier to muscle repair [16]. Animal models showed that sedentary lifestyles impair new muscle fiber formation and prolong inflammatory phases, increasing fibrosis risk. Regular physical activity is essential for maintaining optimal muscle health. In professional soccer players, sport-specific rehabilitation has been shown to outperform PRP injections in reducing muscle injury recurrence rates [17]. While PRP is often used for more severe injuries, targeted rehabilitation protocols remain the gold standard for preventing relapses and optimizing recovery. Whole-body vibration (WBV) has emerged as a promising alternative or complementary exercise strategy [18]. WBV has been shown to improve muscle strength, bone density, and flexibility in both healthy individuals and those with chronic conditions. Personalized protocols are necessary to maximize the benefits of this innovative approach. With aging, reductions in muscle oxygen saturation become increasingly significant [19]. Aging affects oxygen delivery and utilization during exercise, with slower post-exercise recovery times. Understanding these changes is essential for designing exercise programs tailored to older adults. Finally, progressive resistance training appears to be a promising rehabilitation strategy for individuals with hypermobile Ehlers-Danlos syndrome (hEDS) and hypermobility spectrum disorders (HSD) [20]. Despite the variability of these genetic conditions, evidence suggests that personalized programs can improve joint function and reduce pain, offering new directions for rehabilitation.

## 3. Conclusions

The research presented in this Special Issue highlights several promising directions for improving the management of musculoskeletal disorders through exercise. A key area of focus is the personalization of exercise protocols, which involves tailoring interventions to individual biomechanical, physiological, and psychological profiles. This approach aims to optimize functional recovery, minimize the risk of injury, and address specific needs such as metabolic management, postural corrections, and muscle activation imbalances. In addition, longitudinal studies are critical for evaluating the long-term effectiveness of interventions such as electrically induced low-force exercise, sport-specific rehabilitation, and whole-body vibration, enabling refinement of these strategies for clinical and home use. The contributions to this Special Issue highlight the potential role of exercise in the management of musculoskeletal disorders and emphasize the importance of continued innovation in this field. We thank all the authors, reviewers, and editors whose efforts have enriched this collection.
